# Propionate Production by Bioelectrochemically-Assisted Lactate Fermentation and Simultaneous CO_2_ Recycling

**DOI:** 10.3389/fmicb.2020.599438

**Published:** 2020-12-15

**Authors:** Marco Isipato, Paolo Dessì, Carlos Sánchez, Simon Mills, Umer Z. Ijaz, Fabiano Asunis, Daniela Spiga, Giorgia De Gioannis, Michele Mascia, Gavin Collins, Aldo Muntoni, Piet N. L. Lens

**Affiliations:** ^1^Department of Civil and Environmental Engineering and Architecture, University of Cagliari, Cagliari, Italy; ^2^Microbiology, School of Natural Sciences and Ryan Institute, National University of Ireland Galway, Galway, Ireland; ^3^Infrastructure and Environment Research Division, School of Engineering, University of Glasgow, Glasgow, United Kingdom; ^4^IGAG-CNR, Environmental Geology and Geoengineering Institute of the National Research Council–Piazza D’Armi 1, Cagliari, Italy; ^5^Dipartimento di Ingegneria Meccanica, Chimica, e dei Materiali, Università degli Studi di Cagliari, Cagliari, Italy

**Keywords:** bioelectrochemical systems, cyclic voltammetry, electrofermentation, lactate fermentation, microbial electrosynthesis, miseq sequencing, propionate production

## Abstract

Production of volatile fatty acids (VFAs), fundamental building blocks for the chemical industry, depends on fossil fuels but organic waste is an emerging alternative substrate. Lactate produced from sugar-containing waste streams can be further processed to VFAs. In this study, electrofermentation (EF) in a two-chamber cell is proposed to enhance propionate production *via* lactate fermentation. At an initial pH of 5, an applied potential of −1 V vs. Ag/AgCl favored propionate production over butyrate from 20 mM lactate (with respect to non-electrochemical control incubations), due to the pH buffering effect of the cathode electrode, with production rates up to 5.9 mM d^–1^ (0.44 g L^–1^ d^–1^). Microbial community analysis confirmed the enrichment of propionate-producing microorganisms, such as *Tyzzerella* sp. and *Propionibacterium* sp. Organisms commonly found in microbial electrosynthesis reactors, such as *Desulfovibrio* sp. and *Acetobacterium* sp., were also abundant at the cathode, indicating their involvement in recycling CO_2_ produced by lactate fermentation into acetate, as confirmed by stoichiometric calculations. Propionate was the main product of lactate fermentation at substrate concentrations up to 150 mM, with a highest production rate of 12.9 mM d^–1^ (0.96 g L^–1^ d^–1^) and a yield of 0.48 mol mol^–1^ lactate consumed. Furthermore, as high as 81% of the lactate consumed (in terms of carbon) was recovered as soluble product, highlighting the potential for EF application with high-carbon waste streams, such as cheese whey or other food wastes. In summary, EF can be applied to control lactate fermentation toward propionate production and to recycle the resulting CO_2_ into acetate, increasing the VFA yield and avoiding carbon emissions and addition of chemicals for pH control.

## Introduction

The global chemical industry production capacity nearly doubled from 2000 to 2017, increasing from 1.2 to 2.3 billion tons (UNEP, 2019). Chemical production still largely depends on fossil fuels, consuming about 600 Mt of oil and 105 billion Nm^3^ of natural gas as feedstock annually (International Energy and Agency, 2018). Among industries, the chemical sector is the third-largest emitter of greenhouse gases worldwide, releasing about 2 Gt CO_2_eq annually (International Energy and Agency, 2018). However, the increasing price of crude oil and stringent legislation regulating waste management and CO_2_ emissions are expected to drive a shift toward bio-based chemical production (Alibardi et al., 2020). Several chemicals can be produced biologically from organic substrates, including waste feedstocks. Among biological waste treatment processes, fermentation can link waste treatment and chemicals production, converting organic contaminants to valuable products, such as carboxylic acids for use as building blocks in synthesizing a wide range of other chemicals (Atasoy et al., 2019).

Considering carboxylic acids, propionic acid has a higher market value (1.8–2.3 € kg^–1^) than butyric (1.4–1.6 € kg^–1^) and acetic (0.4–0.7 € kg^–1^) acid (Grand View Research, 2015, 2020; Markets and Markets, 2015; Asunis et al., 2020). Its market is expected to expand at an annual rate of 3.5% up to 2026 (Allied Market Research, 2020) due to its diverse applications, such as in grain and food preservation, herbicide and cellulose acetate propionate (CAP) synthesis, and as intermediate for the pharmaceutical and perfume industries (Liu et al., 2012). Moreover, the use of propionic acid in emerging sectors, e.g., as a precursor for biopolymers production (Larsson et al., 2016; Tebaldi et al., 2019), is a further driver for market expansion. Propionic acid is currently mainly produced by petrochemical processes, whose competitiveness is strictly linked to the price of oil (Ahmadi et al., 2017). Sustainable and low-cost processes, such as fermentation of waste feedstocks, offer interesting alternatives, reducing pressure on non-renewable resources and allowing for de-coupling of propionic acid production from oil market dynamics.

To date, fermentative propionate production has mainly relied on pure *Propionibacterium* cultures (Ahmadi et al., 2017) *via* either direct reduction or the dicarboxylic acid pathway (Murali et al., 2017). Lactose, or its main fermentation product, lactate, are used as carbon source. In direct reduction, also known as the acrylate pathway, lactate is reduced to propionate with acryloyl-CoA as intermediate (Akedo et al., 1983). In the dicarboxylic acid pathway, lactate is first converted to succinate, and then to propionate *via* decarboxylation (Paynter and Elsden, 1970). In both cases, acetate and CO_2_ are produced as by-products. Li et al. (2016) showed lactate as a crucial intermediate in fermenting organic waste to propionate, obtaining a maximum concentration of 145 mM (68.3% of total VFAs) with a yield of 0.59 mol mol^–1^ lactate and productivity of 97 mM d^–1^. However, when organic waste is used as carbon source, a two-stage fermentation process is typically required in which the feedstock is first hydrolysed and fermented into a lactate-rich broth, which is then sterilized and fed to pure cultures of propionate-producing microorganisms (Li et al., 2016). Furthermore, dosing of alkaline chemicals is necessary to increase the pH of fermented organic waste toward neutrality for the propionate-producing bacteria (Liu et al., 2012).

Mixed fermentative cultures are, in general, more resilient to the operational fluctuations typical of waste streams, and easier to handle than pure cultures, not requiring sterilization (Wang and Wan, 2009) and representing a low-cost alternative for one-stage propionate production. However, propionate has seldom been reported as the prevalent organic product in mixed-culture fermentation (Lee et al., 2014; Dionisi and Silva, 2016; Strazzera et al., 2018; Asunis et al., 2020). Rather, acetate and butyrate are the soluble products most commonly obtained, especially at pH < 6 (Asunis et al., 2019). Moreover, due to the fast acidification resulting from organic waste fermentation, a substantial quantity of buffer (e.g., sodium hydroxide) may be required for pH control in large-scale reactors.

Electrofermentation (EF), in which a solid electrode acts as a source of oxidizing (anodic EF) or reducing (cathodic EF) power, is a technology recently proposed to overcome the metabolic limitations of fermentative pathways (Schievano et al., 2016). It is recognized that application of current can affect the extracellular and intracellular oxidation-reduction potential (ORP), and thus the metabolic regulations, in fermentative microorganisms (Moscoviz et al., 2016). The electrode can also act as an additional electron source to obtain otherwise energetically unfavorable reactions, and even promote syntrophic interaction between fermenters and electroactive bacteria (Moscoviz et al., 2018). Thus, anodic EF can be applied to dissipate electrons when the substrate is more reduced than the products, e.g., for the conversion of glycerol to ethanol (Speers et al., 2014) or 3-hydroxypropionic acid (Kim et al., 2017), whereas cathodic EF has been applied to synthesize products not commonly obtained in dark fermentation, such as butanol (Engel et al., 2019) or 1,3-propanediol (Xafenias et al., 2015).

Furthermore, cathodic EF presents two additional advantages over dark fermentation. First, proton consumption/OH^–^ generation at the cathode (Grim et al., 2020) provides for cost-effective pH buffering. Even when treating acidic substrates, a micro-environment with higher pH is formed on the electrode surface, that could mitigate the inhibitory effects on the microbial community, and shift the metabolic pathways with respect to dark fermentation. Second, when enough negative potential is applied, CO_2_ produced from fermentation can potentially be recycled into carboxylic acids *via* microbial electrosynthesis (MES) (Nevin et al., 2010). This could result in higher VFA yields and, theoretically, in full recovery as soluble product of the substrate carbon content. Therefore, in this study, cathodic EF was applied to synthetic wastewaters containing lactate, alone or in combination with butyrate to simulate conditions typically achieved in fermented cheese whey (Asunis et al., 2019), which was selected as a model organic substrate. The metabolic shifts compared to dark fermentation, and the possibility of recycling CO_2_ into soluble products *via* MES, were evaluated. The full metabolic pathway was hypothesized based on stoichiometric evaluations, along with the extensive electrochemical and microbiological characterization.

## Materials and Methods

### Microbial Electrochemical Cell Set-Up

**FIGURE 1:**
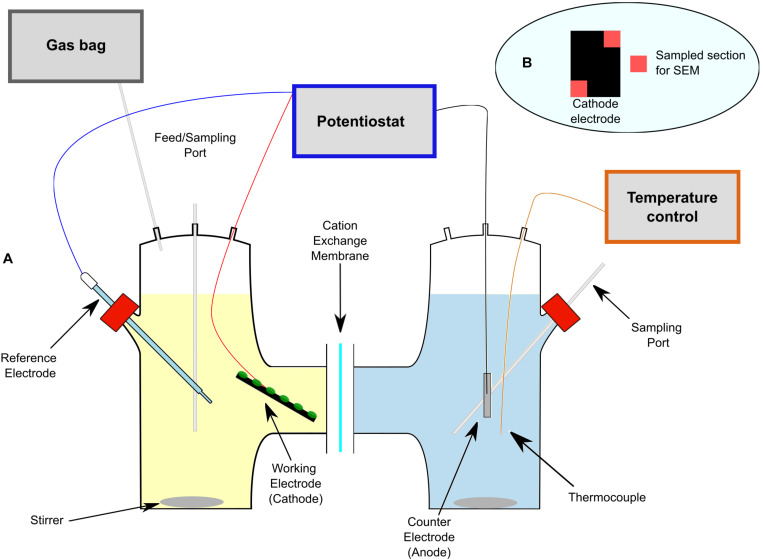
**(A)** Schematic overview of the electrofermentation cells used in this study. **(B)** Section of the cathode electrode showing the location of samples collected for SEM analysis.

The experiments were performed in H-type bioelectrochemical cells (Figure 1), each with a working volume of 150 mL. Two chambers were connected through a circular (3 cm diameter) proton exchange membrane (Nafion 117, Fuel Cell Store, United States, or Fumasep FKE-50, Germany). The Nafion membrane was pre-treated according to Modestra and Mohan (2017). The cathode headspace was connected to a gas bag (1 L) for gas monitoring, and sampling ports were incorporated for both catholyte and anolyte. The cathode (3 × 4 × 0.05 cm) was a carbon cloth (Panex 30 Fabric PW06, Fuel Cell Store, United States), whereas the anode (2 × 2 cm) was a platinized titanium mesh (Goodfellow, United Kingdom). Both electrodes were connected to a potentiostat (VMP3, Biologic, France) using Ti-wire, which was connected to the anode and cathode electrodes by direct contact and through a nylon screw, respectively. Both contacts resulted in a resistance < 5 Ω. An Ag/AgCl reference electrode (BASi RE-5B, Alvatek, United Kingdom) was placed in the cathodic chamber, a few centimeters from the cathode electrode and away from the ion migration path (Harnisch and Freguia, 2012). Temperature control (25 ± 3°C) and stirring were achieved using a hot stirring plate (Cole-Parmer, United States).

### Inoculum, Anolyte, and Catholyte

The inoculum [66.0 ± 3.0 g L^–1^ total solids (TS), 49.8 ± 2.6 g L^–1^ volatile solids (VS)] was sampled from the anaerobic digester of a dairy processing plant (Dairygold, Ireland). Anolyte composition was as follows, expressed in g L^–1^: KH_2_PO_4_ (0.33), K_2_HPO_4_ (0.45), NH_4_Cl (1.0), KCl (0.1), NaCl (0.8), and MgSO_4_ × 7H_2_O (0.2). In addition, the catholyte solution contained 1 mL L^–1^ vitamin solution and 10 mL L^–1^ trace metal solution (DSMZ medium 144). D-lactate, butyrate or both were added to the catholyte, as specified in section “MES reactor operation”. Methanogenic activity was suppressed by adding 0.5 g L^–1^ bromoethanesulphonic acid (BESA) in the first batch cycle.

### MES Reactor Operation

In a first set of experiments, duplicate microbial EF cells (LB1 and LB2) containing both lactate and butyrate (20 mM) were inoculated with 1 g VS L^–1^ digested sludge and operated for four consecutive batch cycles of 5–7 days each. A cathodic potential of −1.2 V vs. Ag/AgCl was imposed on the duplicate cells for the first 24 h of operation, and then reduced to −1 V vs. Ag/AgCl for the remainder of the experiment to prevent an excessive pH increase. Before inoculation, the initial pH of the catholyte was corrected to 5 with 3M NaOH. Catholyte (1 mL) and anolyte (0.5 mL) were sampled each working day for analysis. The decrease in volume of catholyte due to the sample withdrawn for analysis was less than 5% in total. The volume was reintegrated at the beginning of each cycle by addition of fresh medium containing the lactate amount required to restore the initial concentration of 20 mM. Butyrate was only added on the first cycle since it was not consumed by the microbial community. Gas samples were collected from the gas bag for analysis when production was apparent at the end of a batch cycle.

In a second set of experiments, duplicate cells (L1 and L2) were operated with only lactate as the substrate, under the otherwise same operational conditions as in the first set of experiments, for two batch cycles of 8–10 days each. The lactate concentration was 20 mM initially but was increased to 30 mM at the beginning of the second batch cycle to assess the impact of lactate concentration on reactor performance. Finally, a third cell (L3) was operated for one batch cycle with high lactate (150 mM) to simulate concentrations achievable in organic waste fermentation.

Control experiments were included to support the results obtained in the EF studies. A cell with the same characteristics, but without inoculum, served as abiotic control to monitor possible electrochemical reactions, as well as carboxylic acid migration through the membrane. Additionally, non-electrochemical control experiments were performed for one batch cycle to investigate metabolic differences between EF and dark fermentation. Duplicate serum bottles were set up with the same inoculum and solution volume as the EF cells, with lactate and butyrate (C_*LB*_), or only lactate (C_*L*_), as substrate. The initial pH of non-electrochemical control incubations was set at 5 or 7 by dosing 2M NaOH, to distinguish between the effects of the applied potential and pH in the EF experiments.

### Microbiological Analysis

Cathodic and planktonic community samples were collected from LB1 and LB2 cell at the end of the first set of experiments. Cathodes were removed from the cell, and screws and titanium wire were gently disconnected. Sections of 1 × 1 cm were cut from opposite corners of the electrode (see Figure 1) for SEM analysis, under flame, using UV-sterilized instruments and surfaces, and all instruments and surfaces were sterilized with ethanol between two consecutive samples. The remaining part of the electrode was placed in a Falcon tube filled with 5 mL of sterile 0.1 M phosphate-buffered saline (PBS) solution, sonicated at 50–60 Hz and 30% power for 10 min (Bandelin Sonorex Digiplus sonicator), and vigorously vortexed (Fisherbrand ZX3) to detach biofilm. The visible carbon fibers were then removed using sterile tweezers. The cathodic biofilm samples, as well as triplicate sample (5 mL) of catholyte, were then centrifuged at 3,500 rcf for 10 min and resuspended in 3 and 1 mL sterile PBS, respectively. The re-suspended cathodic biofilm was then divided into triplicate 1-mL samples. All samples were snap-frozen in liquid nitrogen and stored at −80°C until further analysis.

DNA was extracted following a chloroform phenol-based extraction method, and 16S rRNA genes were amplified using the primers pair 515F and 806R as previously described (Dessì et al., 2019). The polymerase chain reaction (PCR) protocol included an initial denaturation at 95°C (3 min), followed by 25 cycles of denaturation at 90°C, annealing at 55°C, and extension at 72°C (30 s each). Library preparation and high-throughput sequencing were performed by FISABIO (Valencia, Spain, *fisabio.san.gva.es*) in an Illumina Miseq platform. The sequences generated were deposited in the NCBI Sequence Read Archive (SRA) with accession number PRJNA669689.

Amplicon Sequence Variants (ASVs) were constructed using Qiime2 workflow. In the final analysis, 3,103 clean ASVs were extracted for *n* = 12 samples on which different multivariate statistical analyses were performed using R software. The details of the bioinformatics steps, along with the procedures on the statistical analyses as well as software and R packages used, are provided in Supplementary Material.

### SEM Analysis

Cathode electrode samples were stored in Petri dishes, fixed for 2 h using a solution of glutaraldehyde and paraformaldehyde (2% each) in 0.1 sodium cacodylate buffer pH 7.2. Dehydration was done by passing the samples for two times (15 min each) in an ethanol concentration gradient (30, 50, 70, 90, and 100%), and in hexamethyldisilazane (HMDS). After air drying overnight, the samples were mounted into aluminum stubs with double-sided carbon tabs. The samples were coated with gold using an Emitech K550 sputter coater. Imaging was done using a scanning electron microscope (SEM Hitachi S4700) at an acceleration voltage of 15 kV and 50 μA current.

### Electrochemical Analyses

Chronoamperometric operation and cyclic voltammetries (CVs) were performed using a multi-channel potentiostat (VMP3, Biologic, France) in three-electrode set-up, where the cathode acted as the working electrode. All potential values were reported against the Ag/AgCl reference electrode. Current and cumulative charge values were extracted from the chronoamperometric data using the EC-Lab software. CVs were executed at the beginning and at the end of LB1, LB2, L1, and L2 experiments (without pH modification) between −1.2 and 0 V for four cycles at a scan rate of 1 mV/s. The results reported refer to the third replicate cycle. First derivative analysis of the CV curve was performed using a personalized code on R studio software (Dessì et al., 2021).

### Process Monitoring

Temperature and pH were measured using a thermocouple thermometer (Digi-Sense Temp 10, Cole-Parmer, United Kingdom) and a pH probe (Slimtrode, Hamilton, Switzerland) connected to a controller (Cole Palmer 300, United Kingdom), respectively. Samples from catholyte and anolyte were analysed with a high-performance liquid chromatograph (HPLC) (1260 Infinity II, Agilent, United States) equipped with a Hi-Plex H column held at 60°C and a refractive index detector (RID), using 5 mM H_2_SO_4_ as the mobile phase at a flow rate of 0.7 mL min^–1^. Quantitative analyses were performed to detect carboxylic acids (lactic, acetic, propionic, butyric, valeric, and caproic) and alcohols (ethanol, propanol, and butanol). Only the acids or alcohols concentrations above the detection limit of the instrument were included in the results. Gas composition (H_2_, CH_4_, O_2_, and CO_2_) of the cathode headspace was determined using a gas chromatograph (7890B, Agilent, United States) equipped with a Porapak Q column and a thermal conductivity detector (TCD), with the injection port, oven and detector maintained at 250, 60, and 250°C, respectively.

### Carbon/Electron Balance, Stoichiometric, and EF Performance Calculations

Carbon balances were calculated based on the total moles of carbon fed as lactate or butyrate at the beginning of each batch cycle, and the moles of carbon present as residual substrates or metabolic products (including carboxylic acids and CO_2_) at the end of the experiment. Electron balances (EB) were calculated according to the following equation:

(1) $$\begin{array}{c} EB\left(\%\right)=\frac{\sum_{i=1}^{n}Q_{i}^{out}}{\sum_{j=1}^{m}Q_{j}^{in}+\int_{t0}^{tf}idt} \end{array}$$

where $Q_{j}^{in}$ and $Q_{i}^{out}$ is the charge contained in the carboxylic acids and hydrogen before and after the EF process, respectively, and $\int_{t0}^{tf}idt$ is the charge delivered to the cathode during the experiment.

EF coefficients (η_*EF*_) were calculated as follows (Moscoviz et al., 2016), taking into account only soluble fermentation products:

(2) $$\begin{array}{c} \eta_{EF}=\frac{\int_{t0}^{tf}idt}{\sum_{i=1}^{n}Q_{i}^{out}} \end{array}$$

Theoretical propionate and acetate production was calculated assuming a metabolic pathway that includes lactate fermentation and acetogenesis from electrochemically-produced H_2_ and CO_2_, according to the following equations (Saady, 2013):

Lactate fermentation:

(3) $$\begin{array}{c} 3C_{3}H_{5}O_{3}^{-}\to 2C_{3}H_{5}O_{2}^{-}+C_{2}H_{3}O_{2}^{-}+CO_{2}+H_{2}O \end{array}$$

Acetogenesis:

(4) $$\begin{array}{c} 4H_{2}+2HCO_{3}^{-}+H^{+}\to C_{2}H_{3}O_{2}^{-}+4H_{2}O\left(4\right) \end{array}$$

Production rates were calculated between two consecutive samples as the increment of carboxylic acid concentration divided by the time interval. Production yields were calculated on the whole batch cycle based on the carbon balances between products and substrate. Analysis of variance (ANOVA) was performed to assess significant differences among production rates and yields in the electrofermentation and fermentation experiments at pH 5 and 7. The output of the analysis is provided in Supplementary Material.

Specific energy consumption (E_*C*_) was estimated according to equation (5):

(5) $$\begin{array}{c} E_{C}=\frac{I_{avg}Vt}{\sum_{i=1}^{n}m_{i}}\left(5\right) \end{array}$$

where *m*_*i*_ represents the produced propionic, acetic or butyric acid (in kg) *I*_*avg*_ is the average current during the batch (excluding the start-up with an applied cathodic potential of −1.2 V), *V* is the cell potential (estimated as 3V from punctual measurements in the L3 cell), and t is the duration of the cycle (in hours). The electric power unit cost was estimated based on the average price for industries in Europe (EUROSTAT, 2020).

## Results and Discussion

### Cathodic Electrofermentation of Lactate

#### Microbial Electrofermentation Cell Performance

**FIGURE 2:**
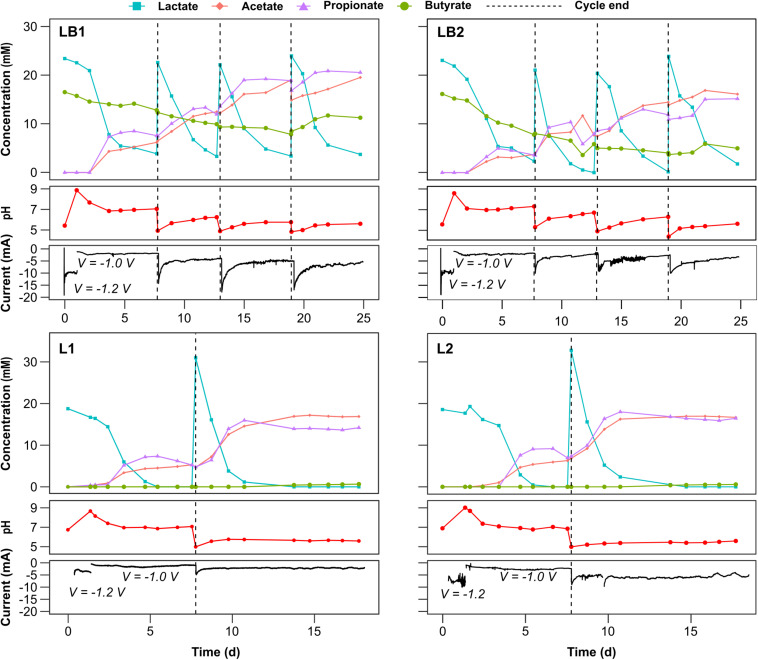
Temporal profiles of carboxylates concentration at the cathode, pH and current profiles for the duplicate cells fed with lactate and butyrate (LB1 and LB2), or in the cells fed with only lactate (L1 and L2). Carboxylates concentration detected at the anode at the end of each batch cycle are reported in Supplementary Table S2.

Imposing a potential of −1.2 V resulted in a current output of about 10 mA in each of the LB1 and LB2 cells (Figure 2). However, the high current caused the pH to increase from an initial value of 5 to 8.6–8.8 in the duplicate cells, and the applied potential was therefore lowered to −1.0 V to avoid further alkalinization of the catholyte. After reducing the potential, the pH returned to 7.7 and 7.1 in LB1 and LB2, respectively, and the current stabilized at around 2.7 and 3.1 mA, compared with currents < 1 mA in the abiotic control (Supplementary Figure S1), suggesting the electrocatalytic activity of biofilm. Two days after reducing the potential, the lactate consumption rate was 8.1 and 4.9 mM d^–1^ in LB1 and LB2, respectively, resulting in propionate and acetate production at maximum rates of, respectively, 4.5 and 2.7 mM d^–1^ (0.33 and 0.16 g L^–1^ d^–1^) in LB1, and 2.0 and 1.4 mM d^–1^ (0.15 and 0.08 g L^–1^ d^–1^) in LB2 on the first batch cycle (Table 1). Such production rates, and yields, are comparable to those obtained in the non-electrochemical control experiment (C_*LB*_1 and C_*LB*_2) at an initial pH of 7, whereas significantly lower yields (below 0.1 mM d^–1^) were obtained in the control incubations at an initial pH of 5 (Supplementary Figure S2 and Table 1). In the three subsequent batch cycles, propionate was produced in both LB1 and LB2 at rates > 1.8 mM d^–1^, despite the decreasing trend of pH, which approached an average value of 5 in the fourth cycle (Figure 2). Since the hydrogen concentration does not affect the propionate-producing lactate fermentation pathway (Seeliger et al., 2002), this suggests that the alkalinization effect of the cathode, rather than the potential applied, triggered lactate conversion into propionate in this study. Cathodic EF can, therefore, be applied to produce propionate from low-pH substrates, mitigating the inhibitory effect of undissociated acids (Van Ginkel and Logan, 2005), with no external addition of bases.

**TABLE 1:** Yields and production rates obtained in each batch experiment.

	Batch	Duration^a^ (h)	Average current^a^ (mA)	Yield (mol mol^–1^ lactate consumed)			Highest production rate (mM d^–1^)		
				Propionate	Acetate	Butyrate^b^	Propionate	Acetate	Butyrate^b^
LB1	I	158.4	2.19	0.39	0.32	−	4.49	2.67	−
	II	120.0	5.39	0.23	0.31	−	2.2	1.71	−
	III	148.8	6.5	0.28	0.36	−	2.76	2.25	−
	IV	139.2	7.68	0.18	0.23	−	1.87	1.00	−
LB2	I	158.4	2.42	0.17	0.18	−	1.98	1.39	−
	II	120.0	3.62	0.21	0.18	−	4.96	3.91	−
	III	148.8	4.51	0.16	0.35	−	2.12	2.81	−
	IV	139.2	5.43	0.19	0.10	−	3.13	1.27	−
L1	I	146.4	1.28	0.28	0.28	0.00	4.74	2.55	0
	II	244.8	2.03	0.30	0.39	0.02	7.2	5.13	0.14
L2	I	146.4	2.08	0.37	0.34	0.00	5.86	2.83	0
	II	244.8	5.44	0.28	0.30	0.02	6.16	4.44	0.12
L3	I	381.6	2.18	0.48	0.30	0.08	12.88	7.22	1.26
C_*LB*_1_pH5	I	285.0	None	0.03	0.10	−	0.04	0.20	−
C_*LB*_2_pH5	I	285.0	None	0.02	0.34	−	0.07	0.48	−
C_*LB*_1_pH7	I	285.0	None	0.44	0.42	−	2.53	1.53	−
C_*LB*_2_pH7	I	285.0	None	0.39	0.42	−	2.40	1.65	−
C_*L*_1_pH5	I	285.0	None	0.25	0.28	0.19	1.67	2.07	0.80
C_*L*_2_pH5	I	285.0	None	0.16	0.37	0.22	0.82	1.15	0.96
C_*L*_1_pH7	I	285.0	None	0.36	0.37	0.00	2.78	1.47	0.09
C_*L*_2_pH7	I	285.0	None	0.45	0.36	0.01	3.94	2.08	0.08

*For EF experiments, the data reported include carboxylates detected in the catholyte and anolyte. C_*LB*_ and C_*L*_ refer to the non-electrochemical control experiments with lactate and butyrate, or only lactate, as substrate, respectively.*

*^*a*^The start-up period at −1.2 V applied potential was excluded.*

*^*b*^Butyrate yield and production rate are not included for LB1, LB2, C_*LB*_2, or C_*LB*_2 because butyrate was added at the beginning of the experiment.*

The current increased with subsequent successive cycles, and the highest average currents of 7.68 and 5.43 mA in LB1 and LB2, respectively, were achieved in the fourth batch cycle (Table 1). Since a similar average pH of 5.2–5.3 was obtained in the fourth batch cycle, the higher current in LB1 suggests a more effective development of the cathodic microbial community than in LB2. Such currents were significantly higher than those obtained in the abiotic control (Supplementary Figure S1), further suggesting its biocatalytic origin. Propionate and acetate production *via* lactate fermentation occurred in all the batch cycles (Figure 2). In LB1, the highest propionate and acetate production rates were achieved in the first batch cycle, and in the subsequent three cycles propionate was produced at a lower maximum rate of 1.9–2.8 mM d^–1^ (0.14-0.21 g L^–1^ d^–1^) up to a cumulative concentration of 20.8 mM, whereas acetate was produced at 1.0–2.3 mM d^–1^ (0.06–0.14 g L^–1^ d^–1^) up to a cumulative concentration of 19.5 mM (Figure 2 and Table 1). The declining trend of production rates observed in the final batch cycle may be attributed to end-product inhibition (Liang et al., 2012) or to biofilm degradation, although the first hypothesis appears more likely, since propionate fermentation is an energetically favorable reaction (Mockaitis et al., 2012) not requiring an electron-donating cathode. This may be mitigated by extracting VFAs before reaching concentrations inhibitive of the microbial community (Jones et al., 2017).

Although the lactate consumption rate was higher in LB2 than in LB1 (11.50 against 7.50 mM d^–1^), similar propionate and acetate production rates were achieved in both. However, the final concentrations, as well as the yields, were even lower in LB2 than in LB1 (Figure 2 and Table 1). This was possibly attributed to the higher O_2_ intrusion from the anodic to the cathodic chamber in LB2, causing a share of carboxylates being consumed by aerobic metabolism. Indeed, at the end of each batch cycle an average of 0.6 mmol O_2_ was found in LB2 headspace, against 0.1 in LB1 (Supplementary Table S1). Interestingly, in LB1 lactate was not completely consumed, but propionate production ceased when the lactate concentration declined below 6 mM. On the other hand, lactate was further consumed in LB2, likely by aerobic metabolism. In both cells, and particularly in LB1, the acetate concentration continued to increase after lactate concentration stabilized, suggesting that a share of acetate was produced through an alternative acetogenic pathway. Since no CO_2_ was detected in the headspace, as would be expected according to Eq. 3, it was likely consumed for the growth of autotrophic organisms at the electrode, and converted into acetate together with (bio)electrochemically-produced H_2_ (Eq. 4) (Nevin et al., 2010).

In the first three batch cycles of the first set of experiments, butyrate showed a linear depletion with a rate of 0.45 and 0.67 mM d^–1^ in LB1 and LB2, respectively, until the conclusion of the third batch cycle. The same trend was observed, with a rate of 0.35 mM d^–1^, in the abiotic control (Supplementary Figure S1), suggesting butyrate migration to the anodic chamber through the membrane. A share of butyrate was likely consumed by aerobic metabolism, particularly in LB2 (Figure 2). Interestingly, from the beginning of the fourth batch cycle, the butyrate concentration in LB1 increased from 8.5 to 11.7 mM, suggesting the onset of the chain elongation pathway (Wu et al., 2020). The same phenomenon occurred in LB2, but was less evident, likely due to concomitant butyrate production and consumption by aerobic metabolism. Caproate production from lactate and butyrate, reported by previous fermentation studies at butyrate concentrations of 35–50 mM (Nzeteu et al., 2018; Contreras-Dávila et al., 2020), was not achieved in the present study, where the butyrate concentration was only 20 mM.

#### Effect of Substrate Concentration

In the first batch of the second set of experiments, when only lactate (20 mM) was provided as the carbon source, maximum propionate production rates of 4.7 and 5.9 mM d^–1^ (0.35 and 0.44 g L^–1^ d^–1^), and acetate production rates of 2.6 and 2.8 mM d^–1^ (0.16 and 0.17 g L^–1^ d^–1^), were achieved in L1 and L2, respectively (Table 1). Such production rates are similar, or slightly higher, than those obtained in LB1, confirming that butyrate was not involved in the fermentation process (Figure 2). Notably, increasing the lactate concentration to 30 mM in the second batch cycle positively impacted the fermentation process, resulting in faster lactate consumption (from 5.7 to 13.6 mM d^–1^ in L1, and from 6.0 to 13.7 in L2), and higher rates of both propionate (7.2 and 6.2 mM d^–1^, or 0.53 and 0.46 g L^–1^ d^–1^, in L1 and L2, respectively) and acetate production (5.1 and 4.4 mM d^–1^, or 0.31 and 0.26 g L^–1^ d^–1^, in L1 and L2, respectively). Butyrate was detected in both cells from day 13 onwards, reaching final concentrations of 0.65 and 0.58 mM in L1 and L2, respectively, suggesting the onset of elongation pathways, as had occurred in the LB1 and LB2 cells. In the non-electrochemical controls, the maximum propionate production rate was 3.9 mM d^–1^ (0.29 g L^–1^ d^–1^) in C_*L*__2_ at an initial pH of 7 (Table 1). However, in the control incubations at pH 5, butyrate was initially produced by lactate fermentation, with the onset of propionate production only 2 days later, when pH rose above 5.5 (Supplementary Figure S2). On average, the propionate production rates in the control incubations at initial pH 5 were significantly lower than those obtained in the EF cells, even after the pH raised above 5.5, whereas no significant differences were obtained between EF cell and control incubations at initial pH 7 (Supplementary Material 4). This confirms that cathodic EF can be applied to trigger propionate production at pH values that are typically more favorable for butyric acid production in dark fermentation.

Since increasing lactate concentrations positively affected propionate and acetate production, a third cathodic EF cell (L3) was fed with 150 mM lactate, which is a concentration obtained in mixed-culture fermentation of carbohydrate-rich substrates, including cheese whey (Tang et al., 2016; Luongo et al., 2019; Pagliano et al., 2019; Dessì et al., 2020). After 2 days start-up, lactate was converted to propionate and acetate, confirming the reproducibility of the process under different lactate loading. On days 2–9, lactate was consumed at an average rate of 12.9 mM d^–1^, similar to the rate achieved in L1 and L2 when feeding 30 mM lactate. An average current of 2.2 mA was detected at an applied potential of −1V, which was lower than in the previous experiements and suggested only a minor role for the electrogenic community, possibly inhibited by the high carboxylate concentrations. The high lactate concentration (150 mM) may have inhibited the acetogenic community in L3 although, to the best of our knowledge, no direct studies on the inhibitory effects of lactate on acetogenic communities are available. This is also confirmed by the lower electrofermentation coefficient (η_*EF*_) obtained in L3 compared with all experiments with a lower lactate concentration (Table 2).

**TABLE 2:** Carbon and charge balances of the cathodic electrofermentation experiments, considering carboxylates detected in both the cathodic and anodic chamber, and gas products in the cathode headspace.

	Inlet (mmol)					Outlet (mmol)										Balance (%)		η_*EF*_
	Lactate		Butyrate		Current	Lactate		Acetate		Propionate		Butyrate		H_2_	CO_2_			
	C	e^–^	C	e^–^	e^–^	C	e^–^	C	e^–^	C	e^–^	C	e^−−^	e^–^	C	C	e^–^	
LB1	36.6	146.5	9.9	49.5	123.8	2.5	9.9	7.9	31.6	12.8	59.6	9.0	45.0	71.0	0.7	70.6	67.9	1.36
LB2	38.6	154.3	9.7	48.4	94.1	2.5	9.9	8.8	35.2	11.5	53.4	5.5	27.7	0.0	0.2	58.9	42.5	1.06
L1	22.3	89.4	0.0	0.0	34.7	0.0	0.0	5.5	22.0	6.8	31.5	0.4	2.0	n.d.^*a*^	n.d.	56.6	44.7	0.63
L2	23.0	92.2	0.0	0.0	83.9	0.0	0.0	5.6	22.2	8.0	37.1	0.3	1.7	n.d.	n.d.	60.1	34.7	1.38
L3	66.9	267.5	0.0	0.0	36.9	7.6	30.3	12.4	49.7	29.1	135.8	7.3	36.4	n.a.^*b*^	n.a.	84.3	82.8	0.17
C_*LB*_1_pH5	9.0	36.0	11.4	57.0	0.0	6.4	25.8	0.2	0.7	0.1	0.3	10.2	51.0	n.d.	n.d.	82.9	83.7	n.a.^b^
C_*LB*_2_pH5	8.8	35.2	11.0	54.9	0.0	2.9	11.6	1.3	5.4	0.1	0.5	10.7	53.3	n.d.	n.d.	76.0	78.7	n.a.^b^
C_*LB*_1_pH7	9.0	36.0	12.0	59.8	0.0	0.1	0.2	2.5	10.0	3.9	18.2	11.1	55.4	n.d.	n.d.	83.7	87.5	n.a.^b^
C_*LB*_2_pH7	9.2	36.7	12.0	59.8	0.0	0.1	0.3	2.5	10.1	3.5	16.5	9.5	47.3	n.d.	n.d.	73.9	77.0	n.a.^b^
C_*L*_1_pH5	9.0	36.0	0.0	0.0	0.0	1.0	3.9	1.5	6.0	2.0	9.4	2.0	10.0	n.d.	n.d.	72.4	81.7	n.a.^b^
C_*L*_2_pH5	8.9	35.5	0.0	0.0	0.0	0.1	0.3	2.2	8.7	1.4	6.6	2.6	13.0	n.d.	n.d.	70.7	80.7	n.a.^b^
C_*L*_1_pH7	8.5	34.2	0.0	0.0	0.0	0.0	0.2	2.1	8.4	3.0	14.2	0.0	0.0	n.d.	n.d.	60.5	66.5	n.a.^b^
C_*L*_2_pH7	9.2	36.9	0.0	0.0	0.0	0.1	0.2	2.2	8.7	4.1	19.3	0.1	0.5	n.d.	n.d.	70.2	77.9	n.a.^b^

*Electrofermentation coefficients (η_*EF*_) were calculated according to Moscoviz et al. (2016). The lower η_*EF*_, the lower is the contribution of microbial electrosynthesis to the electrofermentation process.^*a*^Not detected.^*b*^Not available.*

**FIGURE 3:**
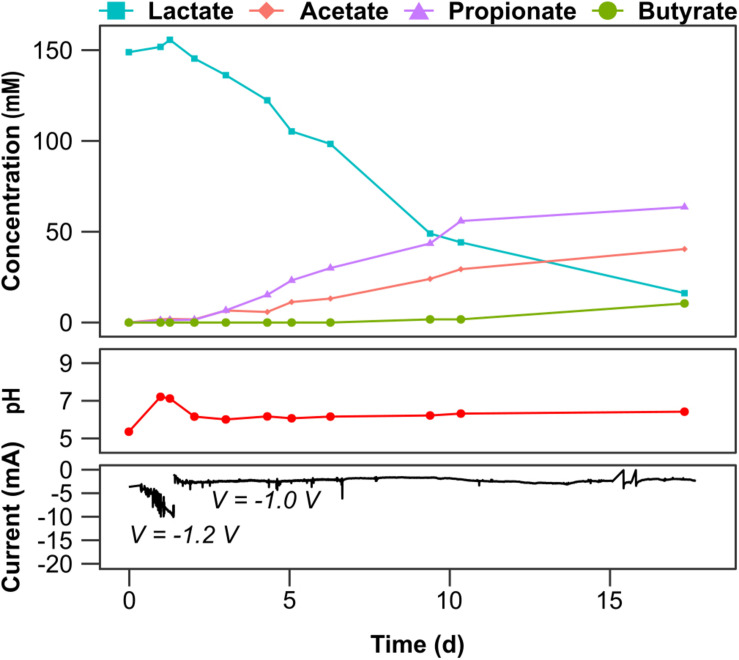
Temporal profiles of carboxylates concentration at the cathode, pH and current profiles for cell L3 fed with 150 mM lactate. Carboxylates concentration detected at the anode at the end of each batch cycle are reported in Supplementary Table S2.

Propionate and acetate were produced in L3 at an average rate of 5.7 and 2.8 mM d^–1^ (0.42 and 0.17 g L^–1^ d^–1^), with peaks of 12.9 and 7.2 mM d^–1^ (0.96 and 0.43 g L^–1^ d^–1^), respectively. Butyrate was also produced from day 9 onward, at an average rate of 1.1 mM d^1^ (0.10 g L^–1^ d^–1^). However, lactate consumption (4 mM d^–1^), and propionate and acetate production (1.6 and 1.1 mM d^–1^, respectively), was slower from day 10 (Figure 3), as a response to VFA accumulation. Notably, a propionate yield of 0.48 mol mol^–1^ lactate consumed was achieved in L3, substantially higher than the yield achieved at lower lactate concentrations (Table 1). The energy invested for the EF process was < 1 kWh kg^–1^ VFA_*produced*_ that, considering the EU-27 average industry price for electricity of 0.1173 € kWh^–1^, further highlights its potential for industrial applications.

#### Carbon and Electron Balances

Carbon and electron balances (Table 2) showed that, when both lactate and butyrate were supplied as substrates, about 70.6% of the carbon and 67.9% of the electrons supplied both as chemicals or electric current were recovered as soluble products or residual lactate in LB1. The remaining carbon was used for microbial growth or diffused outside the cell as CO_2_ from the anodic chamber. Carboxylic acid migrating to the anodic chamber could indeed have been electrochemically oxidized due to the positive potential (around 2 V) at the anode. When Pt-containing electrodes are used, such high potential can result in the formation of PtO_*X*_, which has a high reactivity toward organics (Comninellis and Pulgarin, 1991). A share of electrons was also likely consumed for aerobic metabolism as suggested by the electron balance since only 42.5% of the potential charge was recovered in LB2 compared to 70.6% in LB1. Slightly higher carbon and electron recoveries were achieved in the control incubations (Table 2), supporting the conclusion that gas diffusion and oxygen intrusion may have affected the carbon balances of the electrochemical cells. When only lactate was supplied as substrate, 56.6–60.1% of the carbon, and 34.7–44.7% of the electrons, were recovered as products. However, when the lactate concentration was increased to 150 mM, as high as 84.3% of the carbon, and 82.8% of electrons, consumed as lactate were recovered as EF products (acetate, propionate or butyrate) suggesting that, once the microbial community developed, most carbon and electrons were directed toward products, rather than biomass generation. The carbon recovery achieved in L3 is remarkable, being higher than the carbon recoveries of 60–70% typically achieved in traditional dark fermentation (Asunis et al., 2019).

In all EF experiments, regardless of the initial lactate concentration, a total of 7.9–13.7 mmol of carbon were missing in the balance (taking into account carbon removed as samples), likely linked to microbial growth. An exception is LB2, in which the unaccounted carbon was higher (21.8 mmol), likely due to aerobic metabolism. Accumulation of polyhydroxyalkanoates should also be taken into account as a possible explanation for carbon loss, since it was already reported in microaerophilic biocathodes (Srikanth et al., 2012). This highlights the fact that, over long-term operation and high substrate concentrations, cathodic EF can result in higher carbon recovery as soluble products than dark fermentation, although oxygen intrusion must be strictly prevented.

### Microbial and Metabolic Dynamics

**FIGURE 4:**
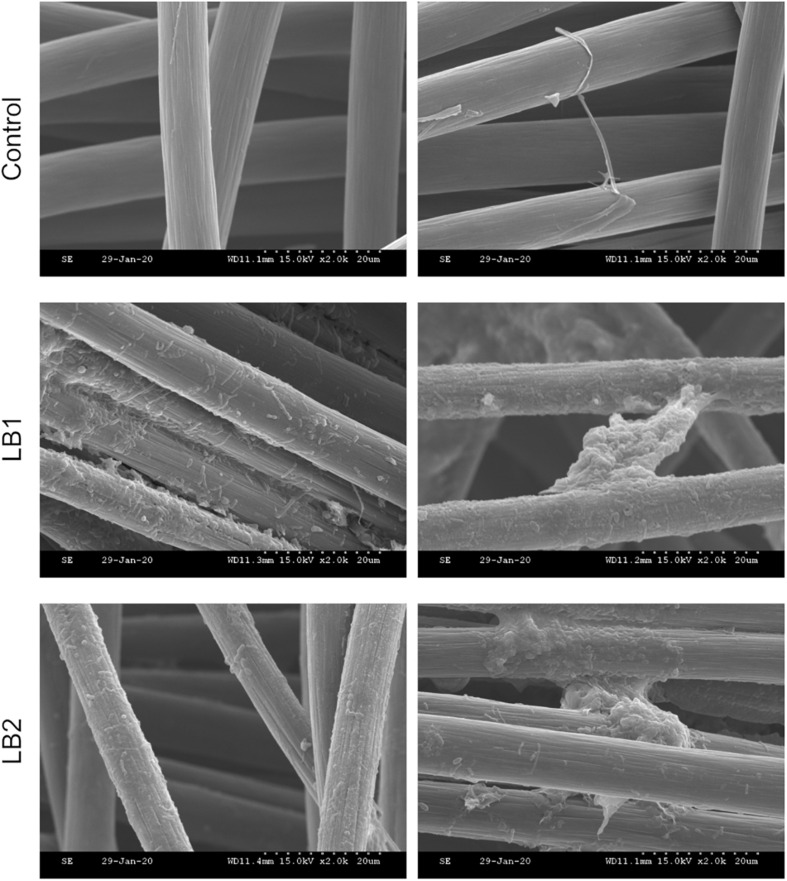
SEM micrographs of the cathodic biofilm from LB1 and LB2 reactors, and in the abiotic control. The magnification is 2,000x.

SEM imaging confirmed the microbial attachment on the cathode of both LB1 and LB2 cells (Figure 4). Single cells attached to the electrodes were detected, as well as more complicated structures developed on the carbon fibers. Interestingly, bacterial structures connecting different carbon fibers were also detected (Figure 4). Alpha diversity analysis (Supplementary Figure S3) revealed that evenness, richness and diversity of the cathode-attached microbial community in LB1 were significantly higher than in the equivalent LB2 community. This suggests that a more diverse, and possibly more resilient, cathodic microbiome developed in LB1, likely promoted by the lower oxygen contamination. In LB1, furthermore, the richness and diversity of the planktonic community were substantially higher than in LB2, suggesting the development of a more diverse fermentative community that resulted in a higher propionate production (Figure 2). Furthermore, principal component analysis (Supplementary Figure S3), using the weighted UniFrac distance metric, along with PERMANOVA, confirmed significant differences in the microbial communities based on cell (*p* = 0.001 ^∗∗∗^) and community type (*p* = 0.001 ^∗∗∗^) (Supplementary Figure S3).

**FIGURE 5:**
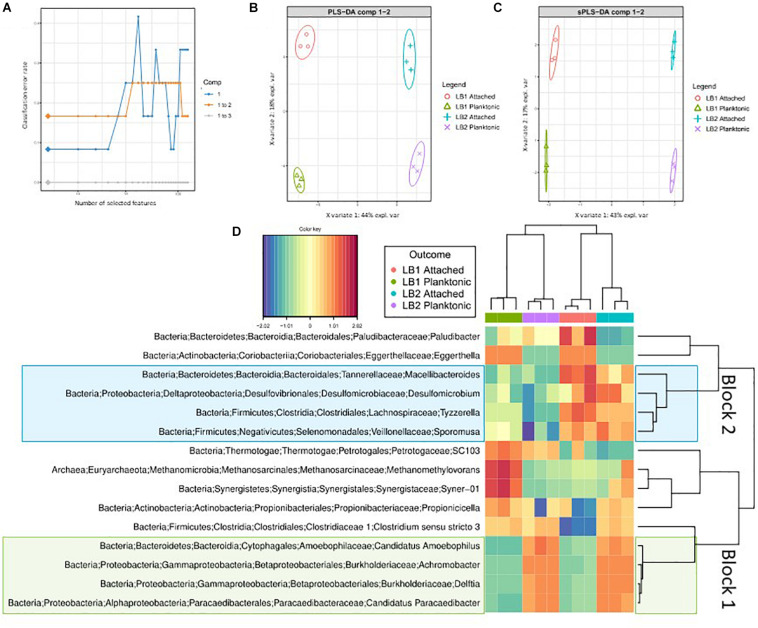
**(A)** sPLS discriminant analysis of amplicon sequencing data. Classification error rates over the components and the numbers of optimal features (genera) in each component, included in the model, chosen for the lowest error rates and represented by diamonds. **(B)** ordination of samples using all the genera in the first two components (sPLS-DA) where ellipses represent 95% confidence intervals and percentage variations explained by these components are denoted in the axes labels. **(C)** Ordination of discriminant taxa from the two components (sPLS-DA). **(D)** Heatmap showing discriminant genera. Rows and columns are ordered using hierarchical (average linkage) clustering. Heatmap depicts TSS + CLR normalized abundances: higher abundances are red and lower abundances are blue.

Sparse Projection to Latent Structure discriminant analysis (sPLS-DA) identified 15 discriminant genera, which accounted for variation between groups. As can be seen in the comparison between attached and planktonic communities in LB1 and LB2, the growth of aerobic species such as *Achromobacter* sp. (Busse and Auling, 2015) and *Delftia* sp. (Sly et al., 2015) in LB2 was the main discriminant between the two cells (Figure 5, Block 1), confirming the impact of oxygen in shaping the community structure. sPLS-DA also identified several genera accounting for most of the differences in microbial community between the attached and planktonic biomass of both cells (Figure 5, Block 2), including *Paludibacter*, *Eggerthella*, *Macellibacteroides*, *Desulfomicrobium*, *Tyzzerella*, and *Sporomusa*. This difference in community structure between the attached and planktonic communities may have been driven by pH changes caused by the buffering capacity of the cathode electrode. Interestingly, within Block 2 *Paludibacter* and *Eggerthella* were more dominant in the attached community of LB1 than LB2. The relatively higher abundance of *Paludibacter*, which are anaerobic propionate-producing organisms (Ueki et al., 2006), in LB1 was likely due to oxygen intrusion to LB2.

**FIGURE 6:**
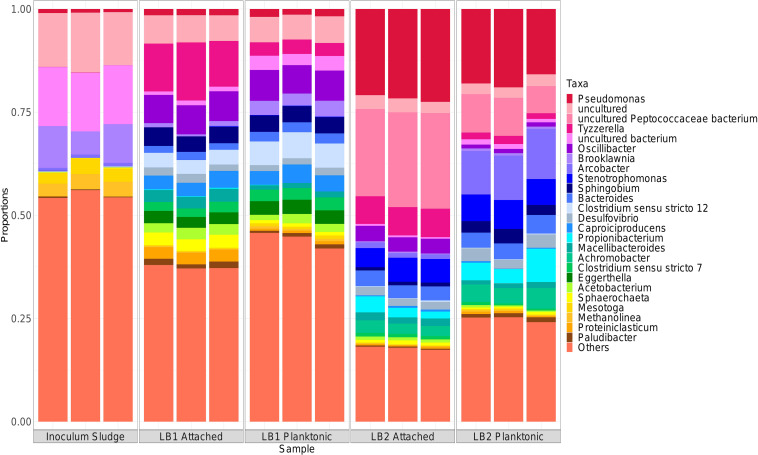
Taxa bars depicting the composition of the cathode-attached and planktonic communities at the conclusion of the experiment with the duplicate cells fed with lactate and butyrate. “Others” represent the sum of the relative abundance of microorganisms outside the top-25.

**FIGURE 7:**
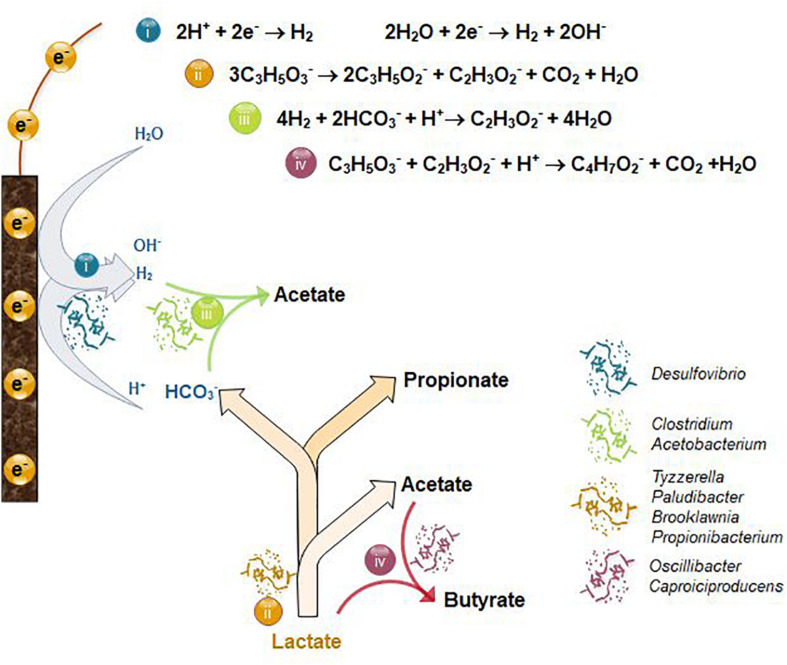
Suggested metabolic pathway for the cathodic electrofermentation of lactate at an applied potential of –1V vs. Ag/AgCl, and the microorganisms involved. The pathway proposed includes (i) (bio)electrochemical hydrogen production, (ii) lactate fermentation, (iii) homoacetogenesis, and (iv) butyrate production by chain elongation.

A higher relative abundance of microorganisms belonging to the order *Clostridiales* was found in both LB1 and LB2, with respect to the inoculum, whereas *Gammaproteobacteria* developed only in LB2 (Supplementary Figure S4). *Gammaproteobacteria* includes several aerobic microorganisms, such as *Pseudomonas* and *Stenotrophomonas* (Palleroni, 2015), which were more relatively abundant in LB2 (Figure 6) and linked to the lower LB2 carboxylate yield compared to LB1. The microbial community in LB1 was indeed composed of anaerobes or facultative anaerobes such as *Clostridium* and *Oscillibacter* (Iino et al., 2007; Figure 6), including taxa involved in propionate production, hydrogen evolution, acetogenesis and chain elongation. Based on the chemical, electrochemical and microbiological data, the likely metabolic pathways occurring in the cathodic electrofermentation cells were hypothesized (Figure 7). Hydrogen evolution at the cathode electrode was likely catalyzed by *Desulfovibrio*, previously identified as part of the core microbiome in electrosynthesis communities and thought to carry out this function (Marshall et al., 2017).

In MES cells, *Desulfovibrio* are typically found in association with autotrophic acetogenic microorganisms such as *Acetobacterium* and *Clostridium* (Marshall et al., 2017; Mateos et al., 2020), where they syntrophically produce acetate from H_2_ and CO_2_ through the Wood-Ljungdahl pathway. Both *Acetobacterium* and *Clostridium* were indeed among the 25 most abundant microorganisms of the LB1 and LB2 communities (Figure 6). The higher relative abundance of both *Clostridium* and *Acetobacterium* in LB1 than in LB2, linked to the lower oxygen concentration, suggests higher acetogenic activity in LB1, which could be linked to the higher current output (Figure 2). Since no CO_2_ was supplied to the cells, acetogenic microorganisms were likely growing syntrophically with fermentative microorganisms producing CO_2_, along with propionate and acetate, from lactate (Figure 7). Interestingly, although a similar lactate fermentation pathway occurred in both cells, the propionate-producing community in LB1 and LB2 was different. *Tyzzerella* sp. were more abundant in LB1 and potentially responsible for most of the propionate production. The *Tyzzerella* genus includes propionate-producing species, such as *T. propionica* (formerly *Clostridium propionicum;* Yutin and Galperin, 2013), and was previously found to be highly abundant in a fermentative reactor converting lactate to propionate and acetate (Xu et al., 2020). Other propionate producers found in LB1 included the facultatively anaerobic *Brooklawnia* (Bae et al., 2006) and *Paludibacter* (discriminant organisms as determined using sPLS-DA). *Propionibacterium* were found in higher relative abundance in LB2 than LB1 in both the cathode-attached and planktonic community. This suggests its role in propionate production in LB2 was facilitated by its optimal growth under microaerophilic conditions. However, in the presence of oxygen, *Propionibacterium* can further oxidize carboxylates to CO_2_ (Koussémon et al., 2001), which could explain the lower propionate and acetate concentrations in LB2 than observed in LB1 (Figure 2).

In all experiments, butyrate was produced when the acetate concentration exceeded 15–20 mM. Butyrate can be produced: (i) electrochemically from CO_2_, H^+^ and electrons from the cathode, (ii) *via* the Acetyl-CoA reductive pathway with H_2_ as electron donor, and (iii) from acetate and lactate, or ethanol, *via* reverse β oxidation (Raes et al., 2017). The last scenario appears most probable in this study, since butyrate production generally occurs concurrently with lactate consumption (Figure 2). In both LB1 and LB2, *Oscillibacter* and *Caproiciproducens* were identified as the butyrate-producing organisms, and their higher abundance in LB1 than LB2 (around 7 and 4%, respectively) (Figure 6) correlated with higher butyrate production in LB1 (Figure 2). Both *Oscillibacter* (Wu et al., 2020) and *Caproiciproducens* (Contreras-Dávila et al., 2020) were previously associated with chain elongation pathways, indicating that, in this study, lactate was the electron donor for butyrate production (Figure 7).

**FIGURE 8:**
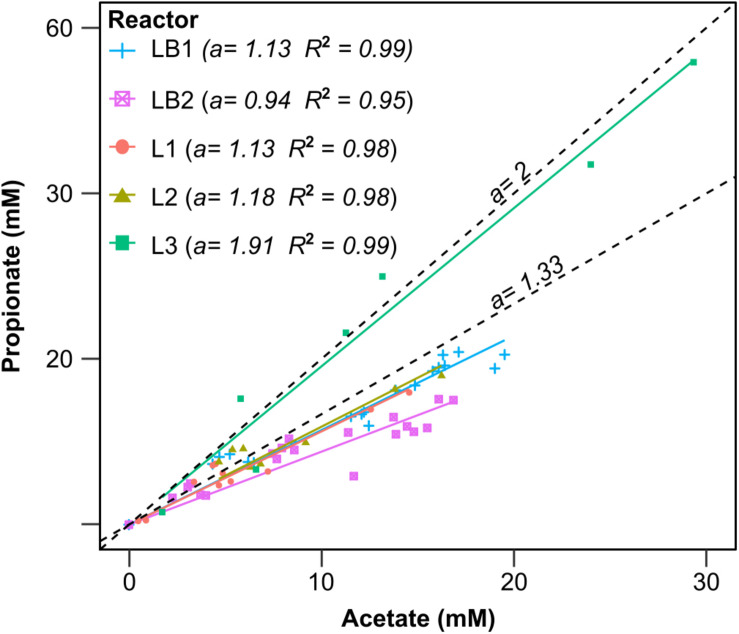
Linear correlation between propionate and acetate concentrations detected in the catholyte of the cells fed with lactate and butyrate (LB1 and LB2), or only lactate (L1, L2, and L3). The data refer to lactate fermentation and acetogenesis only; data collected after the onset of chain elongation pathways, in which butyrate was produced from lactate and acetate, were excluded for simplicity.

According to Figure 7, propionate and acetate are produced from lactate in a molar ratio of 2:1, and an additional mole of acetate is produced from two moles of CO_2_. Thus, when the two reactions occur simultaneously, 0.67 mol propionate and 0.50 mol acetate will be produced from 1 mol lactate, with a propionate:acetate ratio of 1.33. When comparing the experimental results with the theoretical estimations, similar propionate:acetate ratios of 1.13–1.18 were achieved in LB1, L1, and L2 (Figure 8), within a 15% range of the theoretical yield. This confirms that lactate fermentation and acetogenesis occurred simultaneously, and the slightly lower ratio than theoretically predicted can be explained by propionate consumption *via* microaerobic metabolism (Thierry et al., 2011), or acetogenesis. Indeed, a substantially lower propionate:acetate ratio of 0.94 was achieved in the LB2 cell, where more oxygen intrusion occurred (Supplementary Table S1).

Feeding the cell with 150 mM lactate resulted in the highest propionate:acetate ratio of 1.91 (Figure 7). Such a ratio is only 5% lower than the ratio theoretically achieved by lactate fermentation to propionate and acetate, suggesting a minor role of acetogenesis. It is indeed plausible that acetogenic microorganisms were inhibited by the high carboxylate concentrations. Despite this, cathodic EF of lactate resulted in propionate and acetate production with remarkable average rates of 5.6 and 4.6 mM d^–1^, respectively.

### Electrochemical Characterization

**FIGURE 9:**
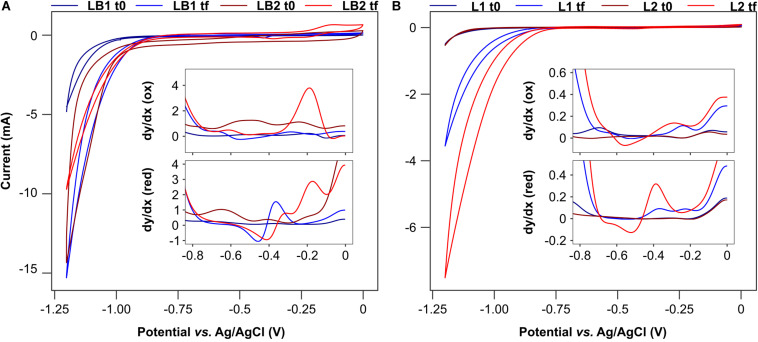
Cyclic voltammetry and first derivative analysis of the cells fed with both lactate and butyrate **(A)**, or only lactate **(B)**.

Cyclic voltammetries (CVs) show a difference of about 0.2–0.3 V between the hydrogen reduction potential at the beginning and at the conclusion of the experiment in LB1, L1 and L2 (Figure 9), which suggests the development of an electroactive biofilm (Labelle et al., 2014). This was less evident in LB2, in which the overpotential was reduced by only 0.1 V. Since a similar final pH of 5.6 was measured in LB1 and LB2 at the end of the experiment (when CVs were performed), this confirms the presence of a weaker electroactive community in LB2, explained by the presence of oxygen, as confirmed by the lower current output in LB2 than in LB1 (Figure 2).

First derivative analysis confirmed the presence of oxidation and reduction peaks at the conclusion of the experiment, whereas flat curves, or small peaks, were detected at the beginning (Figure 9). In LB1, a reversible redox couple was evident, with a reduction peak at −0.24 and the corresponding oxidation peak at −0.29 V, suggesting the presence of reversible redox active molecules at the biofilm-reactor interface (Modestra and Mohan, 2017). A second reduction peak was detected in both LB1 and LB2 at potentials of −0.46 and −0.41 V, respectively. Such a potential is compatible, for example, with cytochromes used by electrogenic bacteria such as *Desulfovibrio* sp. for exchanging electrons with solid electrodes (Yahata et al., 2006). Similar reduction peaks were also detected in L1 and L2, although at lower potential (−0.54 and −0.52 V, respectively), which was attributed to the different biofilm stage than in LB1 and LB2 when the CV analysis was performed (after 2 and 4 batch cycles for L and LB, respectively).

## Conclusion

In this study, electrofermentation is proposed for the valorization of lactate-rich fermentates. An applied potential of −1.0 V vs. Ag/AgCl favored propionate production with a maximum yield of 0.48 mol mol^–1^ lactate consumed obtained with an initial lactate concentration of 150 mM. Furthermore, as confirmed by stoichiometric calculations and microbial community analysis, CO_2_ produced from lactate fermentation was recycled into acetate *via* microbial electrosynthesis, resulting in higher carbon recovery than in dark fermentation, although this process may be inhibited at high lactate concentrations. At an initial lactate concentration of 150 mM, the energy invested for the EF process was < 1 kWh kg^–1^ VFA produced highlighting its potential for application in industry. Further studies on real fermentates, and under continuous operation, will be required to confirm the results obtained here under batch conditions with synthetic feedstocks.

## Data Availability Statement

The datasets presented in this study can be found in online repositories. The names of the repository/repositories and accession number(s) can be found below: NCBI BioProject, accession no: PRJNA669689.

## Author Contributions

MI and PD conceptualized and designed the experiments, and drafted the manuscript. CS and MI realized the script for CV analysis and assisted in its interpretation. SM and UI performed the bioinformatics work and assisted in interpreting the microbiological results. DS, FA, GDG, MM, and AM assisted in results interpretation and revised the manuscript. GC and PL thoroughly revised the final version for submission. All authors contributed to the article and approved the submitted version.

## Conflict of Interest

The authors declare that the research was conducted in the absence of any commercial or financial relationships that could be construed as a potential conflict of interest.
