# Supplementary material for: Propionate Production by Bioelectrochemically-Assisted Lactate Fermentation and Simultaneous CO2 Recycling
Source: Front Microbiol. 2020 Dec 15;11:599438. doi: 10.3389/fmicb.2020.599438 (PMC7769879; doi:10.3389/fmicb.2020.599438)
Supplement: Supplementary file 5 [file Table_5.docx]

Supplementary Material

***SM1. Bioinformatics***

Alpha and Beta diversity analysis were performed using the Vegan package (Oksanen et al., 2015). Alpha diversity was estimated with three measures: (i) *rarefied richness* – an estimate of amplicon sequence variant (ASVs) numbers in a rarefied sample (to minimum library size); (ii) *Shannon entropy* – a widely used and accepted diversity index; (iii) *Pielou’s eveness* – a comparison between actual diversity values and the highest possible diversity value, constrained from 0 to 1.0. Higher values imply less variation in ASV abundance in eachsample. Principal Coordinate Analysis (PCoA) was used to observe beta diversity i.e. differences between samples. ASVs were plotted using the Bray-Curtis distance metric which considers the abundance counts of ASVs. Vegan's betadisper() function was used to analyse multivariate homogeneity of groups. This handles distances between objects and group centroids by reducing the original distances to principal coordinates and reporting significances based on analysis of variance (ANOVA). ANOVA was performed using Vegan’s Adonis() function on distances. This function, referred to as PERMANOVA, fits linear models to distance matrices based on the explanatory variables (meta data) to identify if they can explain the variability in the microbial community between groups.

Differential abundance analysis was performed with the DESeqDataSetFromMatrix() function from DESeq2 package (Love et al., 2014). This identified ASVs, which were significantly different between groups. An adjusted p-value significance cut-off of 0.05 and log2 fold change cut-off of 2 were used. DESeqDataSetFromMatrix() uses negative binomial GLM to get maximum likelihood approxomations for the log fold change of ASVs between groups.Then, Bayesian shrinkage was used to obtain shrunken log fold changes, followed by the Wald test for obtaining significances. Tables containing all data related to log fold changes of ASVs can be found in the “Supplementary Files” folder. ASVs which were significantly different between groups were visualised using differential heat trees (Foster et al., 2017). Heat trees use Wilcox p-value statistics to find nodes that are differentially expressed in compositional microbiome data.

Sparse Projection to Latent Structure – Discriminant Analysis (sPLS-DA) was performed using the MixOmics package (Rohart et al., 2017). Artificial latent components were constructed for predicted variables (ASVs/Genera) and response variables (reactors/communities) by converting these matrices to scores and loading vectors in a new space to get a maximum covariance between the scores of the two matrices. Loading vectors (with piece-wise coefficient for each ASV) were made so that the coefficients indicate how important each variable is in defining the component. Non-zero coefficients for the loading vectors indicate discriminant ASVs by identifying which ones are significantly different between the samples. 1% of ASVs with low counts in the initial ASV table were filtered out to the author’s recommendations given at http://mixomics.org/mixmc/pre-processing/. Subsequently, the ASV table was normalised with Total Sum Scaling (TSS) on the ASVs followed by Centered Log Ratio (CLR) (in conjunction these are referred to as TSS+CLR normalization) before applying the splsda() function. The perf.plsda() and tune.splsda() functions were first used to predict the number of latent components (associated loading vectors) and the number of discriminants by initializing the perf.plsda() procedure with the total number components to be the number of groups used in the study. Next, the first three components were retained as the classification error rates were minimum for these using the centroid distance matrix in the procedure. The tune.splsda() function was then initialized with three components and using leave-one-out cross-validation.

# SM2. Supplementary Figures


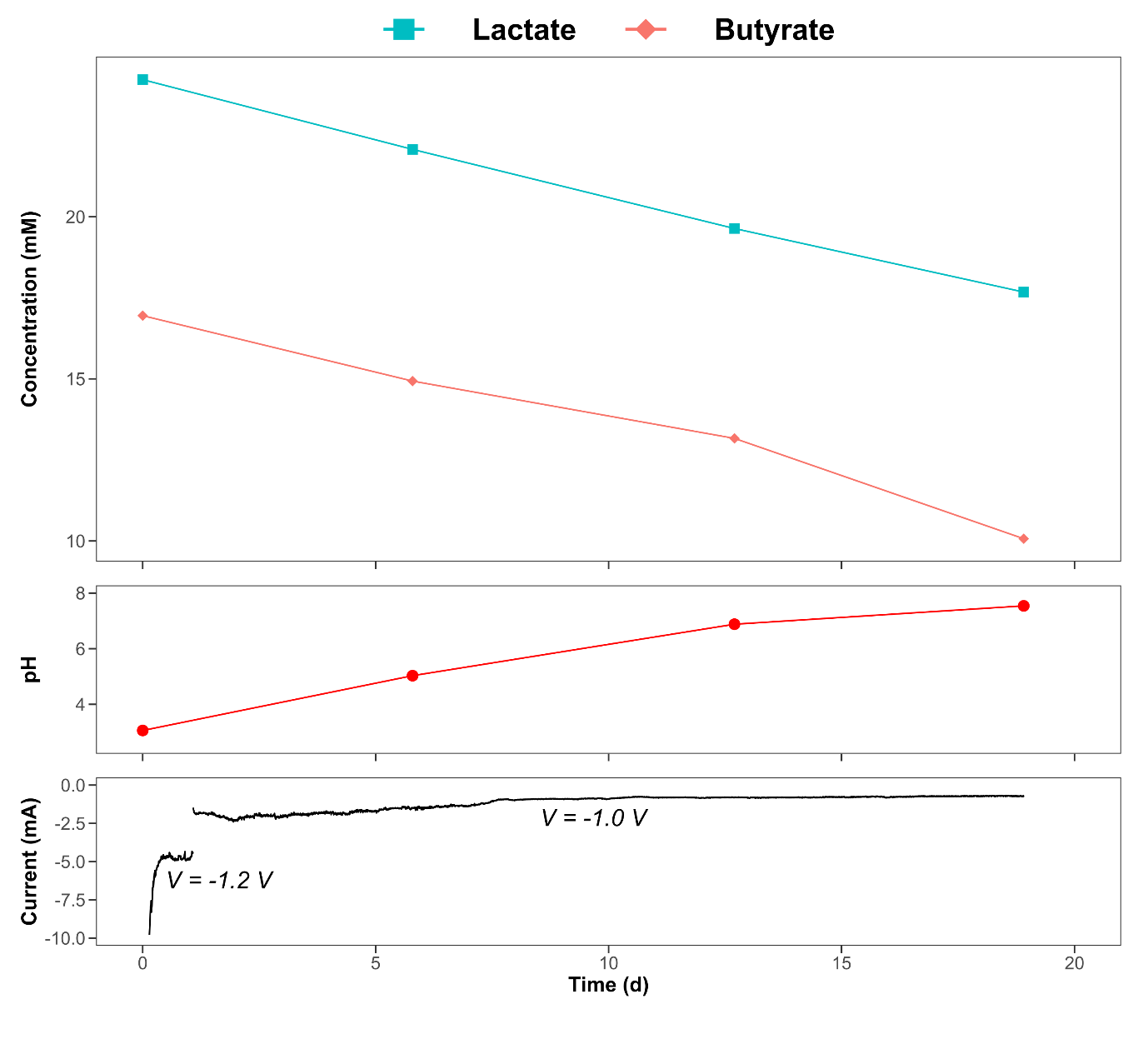
**Figure S1.** Lactate and butyrate concentration, pH and current trend in the abiotic control, highlighting a constant diffusion of both compounds through the Nafion membrane.


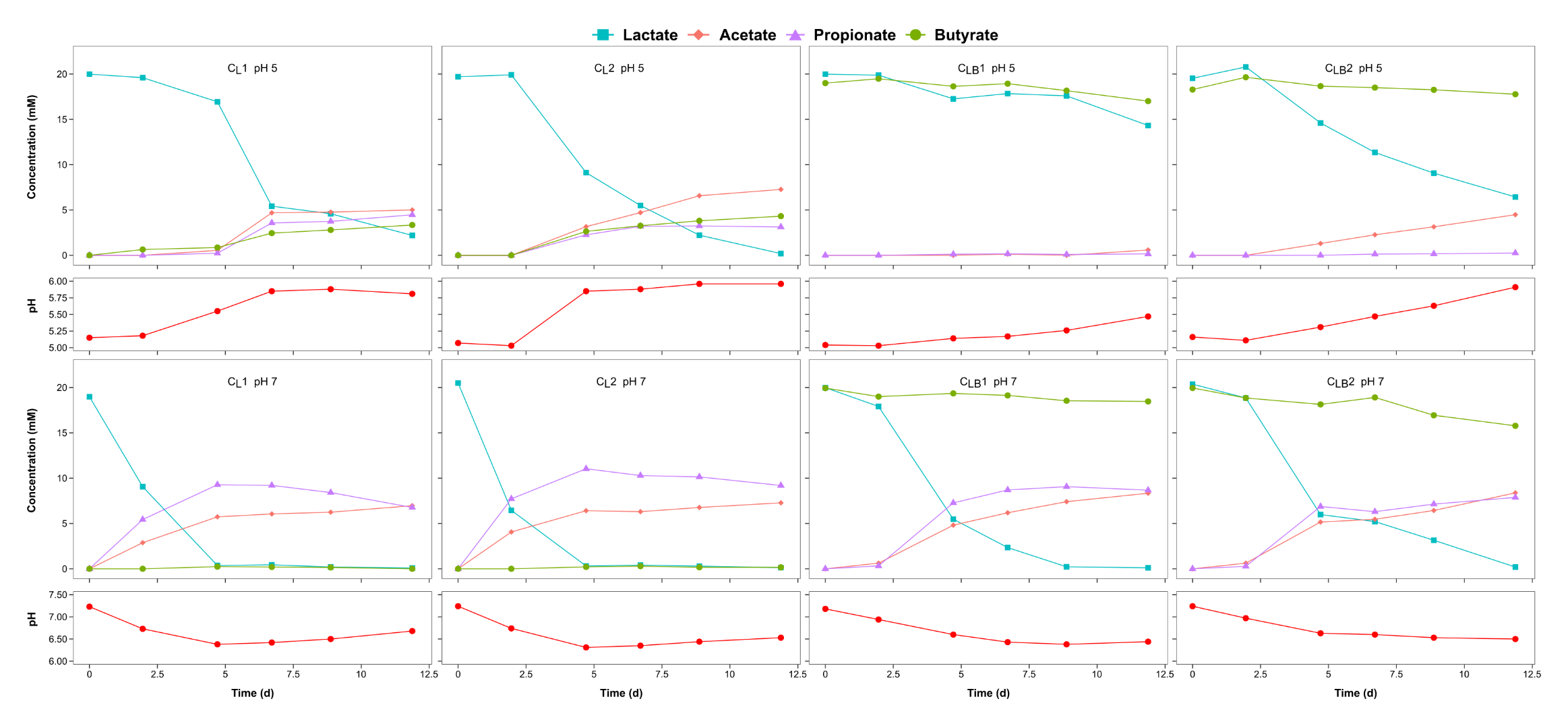


**Figure S2.** Carboxylates concentration and pH profiles over time for the duplicate cells fed with lactate and butyrate (C_LB_1 and C_LB_2), or in the cells fed with only lactate (C_L_1 and C_L_2) at initial pH 5 or 7.


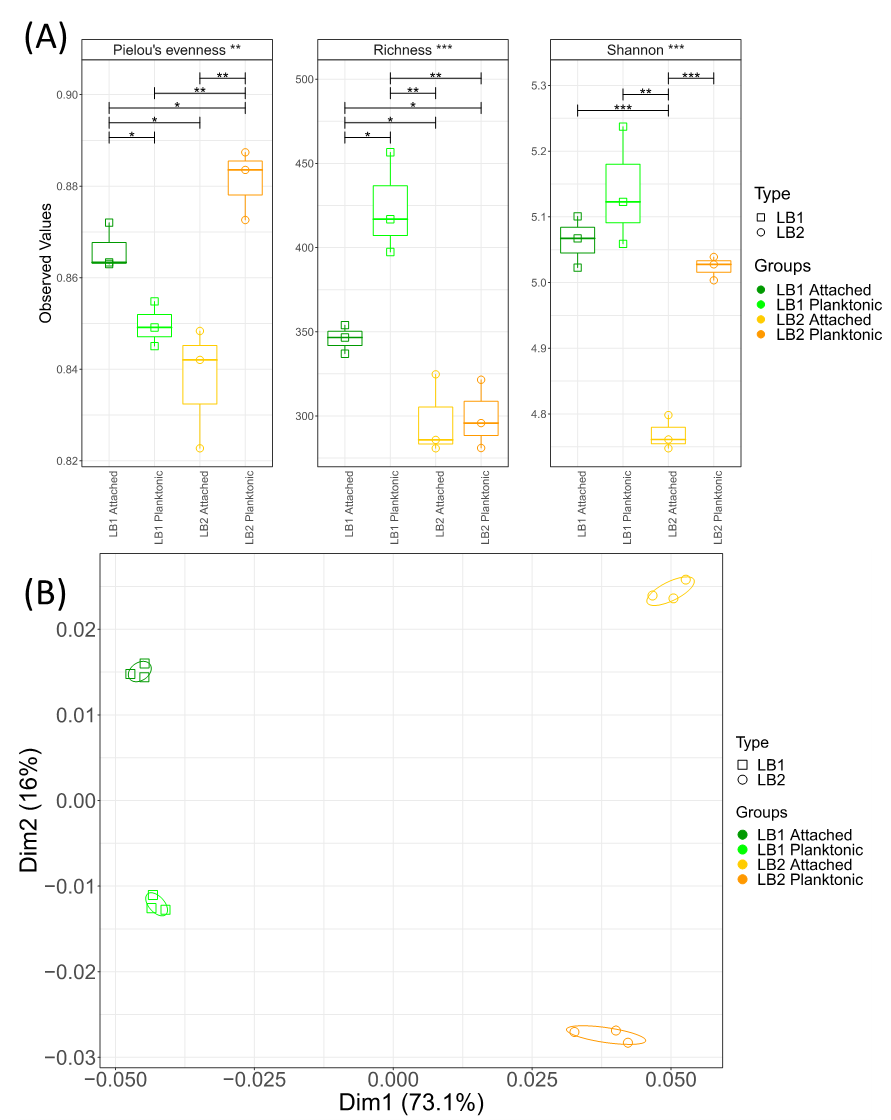


**Figure S3**. (A) Evenness, richness and diversity Shannon diversity indexes. (B) Principal Component Analysis (PCoA) of the cathode attached and planktonic communities in the two replicate cells fed with lactic and butyric acid using the weighted unifrac distance metric.


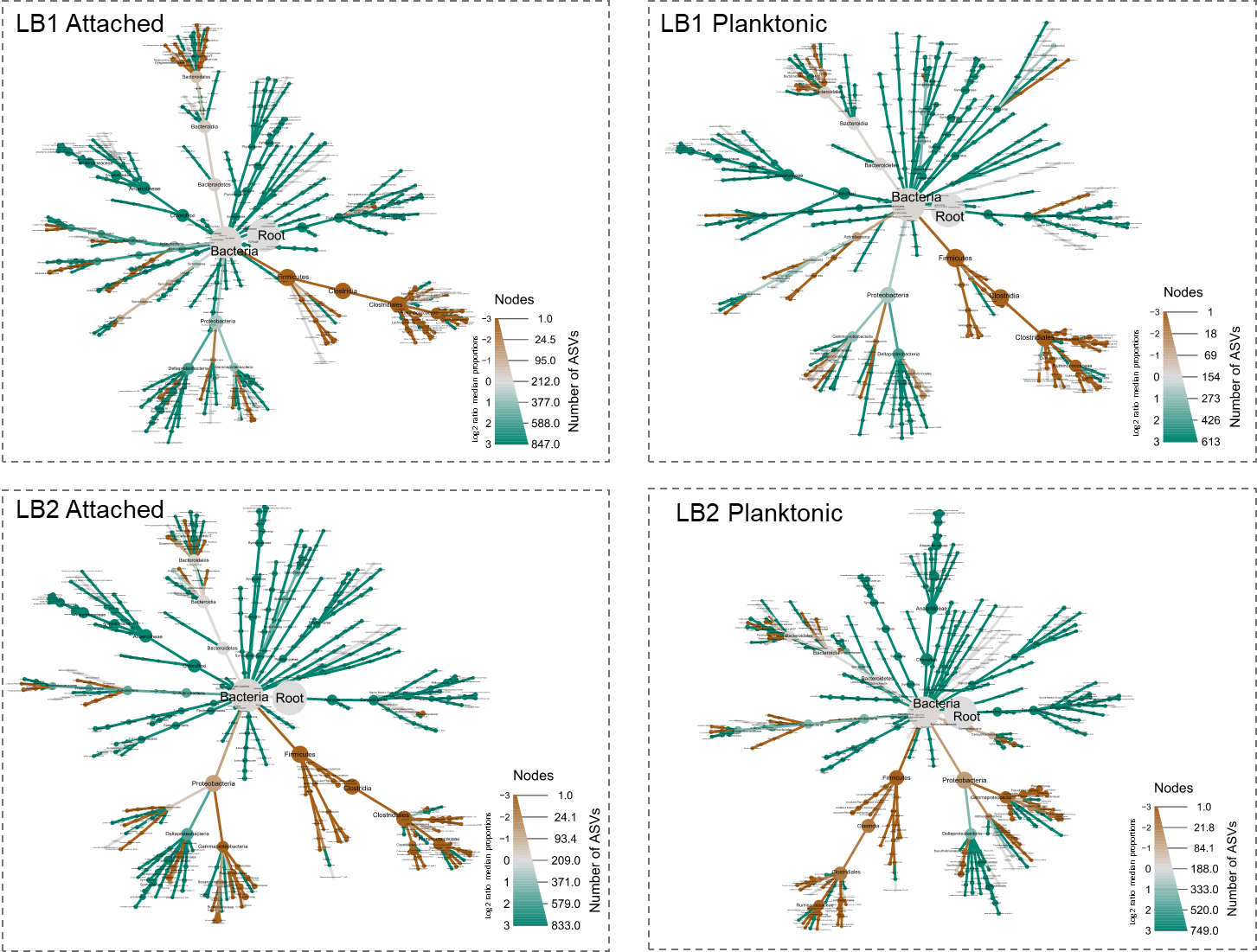


**Figure S4.** Heat trees depicting the microorganisms significantly upregulated in the cathodic and planktonic communities of LB1 and LB2 (brown) with respect to the inoculum (green). ASVs were identified by differential analysis outlined in section SM1 and are available in the Supplementary Tables. These ASVs were then presented as heat trees to observe phylogenetic relatedness. The colour intensity represents the log2 median ratio of proportional abundances i.e. the colour intensity corresponds to the ratio of the relative abundance of a given taxon in the inoculum to its abundance in a given sample, in a log2 scale.

## SM3. Supplementary Tables

**Table S1.** Quantitative and qualitative gas analysis for bioelectrochemical batch experiments with lactate and butyrate as substrates (T= 25 °C, p = 1 atm).

|  | **Batch** | **Gas volume**  **(NmL)** | **H_2_**  **(%)** | **O_2_**  **(%)** | **CO_2_**  **(%)** | **CH_4_**  **(%)** | **H_2_**  **(NmL)** | **O_2_**  **(NmL)** | **CO_2_ (NmL)** | **CH_4_**  **(NmL)** | **H_2_**  **(mmol)** | **O_2_**  **(mmol)** | **CO_2_**  **(mmol)** | **CH_4_**  **(mmol)** |
| --- | --- | --- | --- | --- | --- | --- | --- | --- | --- | --- | --- | --- | --- | --- |
| **LB1** | I | 217.1 | 48.6 | 1.1 | 1.8 | 0.4 | 105.5 | 2.5 | 4.0 | 0.8 | 4.7 | 0.1 | 0.2 | 0.0 |
|  | II | 212.5 | 68.5 | 1.4 | 1.3 | 0.3 | 145.6 | 3.2 | 2.9 | 0.6 | 6.5 | 0.1 | 0.1 | 0.0 |
|  | III | 290.4 | 90.9 | 1.0 | 1.3 | 0.1 | 263.9 | 3.1 | 4.3 | 0.2 | 11.8 | 0.1 | 0.2 | 0.0 |
|  | IV | 282.2 | 99.6 | 1.0 | 1.3 | 0.0 | 280.9 | 3.0 | 4.1 | 0.1 | 12.5 | 0.1 | 0.2 | 0.0 |
| **LB2** | I | 86.6 | 0.5 | 12.3 | 1.8 | 0.1 | 0.5 | 11.6 | 1.7 | 0.1 | 0.0 | 0.5 | 0.1 | 0.0 |
|  | II | 108.1 | 0.0 | 15.0 | 1.3 | 0.0 | 0.0 | 17.6 | 1.5 | 0.0 | 0.0 | 0.8 | 0.1 | 0.0 |
|  | III | 56.8 | 0.0 | 15.2 | 0.1 | 0.0 | 0.0 | 9.4 | 0.0 | 0.0 | 0.0 | 0.4 | 0.0 | 0.0 |
|  | IV | 84.3 | 0.0 | 13.1 | 0.3 | 0.0 | 0.0 | 12.0 | 0.3 | 0.0 | 0.0 | 0.5 | 0.0 | 0.0 |

**Table S2.** Carboxylic acid concentrations detected in the anolyte at the end of each batch cycle.

|  | **Batch** | **Concentration (mM)** | | | |
| --- | --- | --- | --- | --- | --- |
|  |  | **Lactate** | **Acetate** | **Propionate** | **Butyrate** |
| **LB1** | I | 1.91 | 1.82 | 3.78 | 5.01 |
|  | II | 1.45 | 3.08 | 1.85 | 4.20 |
|  | III | 3.90 | 14.95 | 15.99 | 8.97 |
|  | IV | 1.77 | 6.88 | 7.84 | 3.77 |
| **LB2** | I | 1.77 | 1.22 | 2.11 | 4.60 |
|  | II | 1.75 | 3.41 | 2.09 | 5.22 |
|  | III | 2.88 | 11.02 | 9.89 | 5.31 |
|  | IV | 3.73 | 13.22 | 10.31 | 4.26 |
| **L1** | I | 0.00 | 0.00 | 0.21 | 0.00 |
|  | II | 0.00 | 1.50 | 0.85 | 0.00 |
| **L2** | I | 0.00 | 0.00 | 0.27 | 0.00 |
|  | II | 0.00 | 1.88 | 1.26 | 0.00 |
| **L3** | I | 0.68 | 1.02 | 1.18 | 1.64 |

## SM4. Statistical analysis

One-way ANOVA was conducted to assess significant differences in propionic and acetic acid production rate and yield among electrofermentation (EF, including experiments LB1, LB2, L1 and L2), dark fermentation at pH 5 (DF_pH5, including experiments C_LB_1_pH5, C_LB_2_pH5, C_L_1_pH5 and C_L_2_pH5), and dark fermentation at pH 7 (DF_pH7, including C_LB_1_pH7, C_LB_2_pH7, C_L_1_pH7 and C_L_2_pH7). Refer to Table 1 in the manuscript for the production rate and yield values included in the analysis (first batch cycle under each conditions). Results are shown below.

**PRODUCTION RATE**

| **Descriptives** | | | | | | | | | |
| --- | --- | --- | --- | --- | --- | --- | --- | --- | --- |
|  | | N | Mean | Std. Deviation | Std. Error | 95% Confidence Interval for Mean | | Minimum | Maximum |
|  |  |  |  |  |  | Lower Bound | Upper Bound |  |  |
| Acetic | EF | 4 | 2.3600 | .65676 | .32838 | 1.3149 | 3.4051 | 1.39 | 2.83 |
|  | DF_pH5 | 4 | .9750 | .83173 | .41586 | -.3485 | 2.2985 | .20 | 2.07 |
|  | DF_pH7 | 4 | 1.6825 | .27536 | .13768 | 1.2443 | 2.1207 | 1.47 | 2.08 |
|  | Total | 12 | 1.6725 | .82207 | .23731 | 1.1502 | 2.1948 | .20 | 2.83 |
| Propionic | EF | 4 | 4.2675 | 1.63722 | .81861 | 1.6623 | 6.8727 | 1.98 | 5.86 |
|  | DF_pH5 | 4 | .6500 | .76981 | .38490 | -.5749 | 1.8749 | .04 | 1.67 |
|  | DF_pH7 | 4 | 2.9125 | .70292 | .35146 | 1.7940 | 4.0310 | 2.40 | 3.94 |
|  | Total | 12 | 2.6100 | 1.85921 | .53671 | 1.4287 | 3.7913 | .04 | 5.86 |

| **ANOVA** | | | | | | |
| --- | --- | --- | --- | --- | --- | --- |
|  | | Sum of Squares | df | Mean Square | F | Sig. |
| Acetic | Between Groups | 3.837 | 2 | 1.919 | 4.801 | .038 |
|  | Within Groups | 3.597 | 9 | .400 |  |  |
|  | Total | 7.434 | 11 |  |  |  |
| Propionic | Between Groups | 26.722 | 2 | 13.361 | 10.640 | .004 |
|  | Within Groups | 11.302 | 9 | 1.256 |  |  |
|  | Total | 38.023 | 11 |  |  |  |

| **Multiple Comparisons** | | | | | | | |
| --- | --- | --- | --- | --- | --- | --- | --- |
| Tukey HSD | | | | | | | |
| Dependent Variable | (I) Conditions | (J) Conditions | Mean Difference (I-J) | Std. Error | Sig. | 95% Confidence Interval | |
|  |  |  |  |  |  | Lower Bound | Upper Bound |
| Acetic | EF | DF_pH5 | 1.38500^*^ | .44701 | .031 | .1369 | 2.6331 |
|  |  | DF_pH7 | .67750 | .44701 | .329 | -.5706 | 1.9256 |
|  | DF_pH5 | EF | -1.38500^*^ | .44701 | .031 | -2.6331 | -.1369 |
|  |  | DF_pH7 | -.70750 | .44701 | .301 | -1.9556 | .5406 |
|  | DF_pH7 | EF | -.67750 | .44701 | .329 | -1.9256 | .5706 |
|  |  | DF_pH5 | .70750 | .44701 | .301 | -.5406 | 1.9556 |
| Propionic | EF | DF_pH5 | 3.61750^*^ | .79238 | .003 | 1.4052 | 5.8298 |
|  |  | DF_pH7 | 1.35500 | .79238 | .254 | -.8573 | 3.5673 |
|  | DF_pH5 | EF | -3.61750^*^ | .79238 | .003 | -5.8298 | -1.4052 |
|  |  | DF_pH7 | -2.26250^*^ | .79238 | .045 | -4.4748 | -.0502 |
|  | DF_pH7 | EF | -1.35500 | .79238 | .254 | -3.5673 | .8573 |
|  |  | DF_pH5 | 2.26250^*^ | .79238 | .045 | .0502 | 4.4748 |
| *. The mean difference is significant at the 0.05 level. | | | | | | | |

| **Acetic** | | | |
| --- | --- | --- | --- |
| Tukey HSD^a^ | | | |
| Conditions | N | Subset for alpha = 0.05 | |
|  |  | 1 | 2 |
| DF_pH5 | 4 | .9750 |  |
| DF_pH7 | 4 | 1.6825 | 1.6825 |
| EF | 4 |  | 2.3600 |
| Sig. |  | .301 | .329 |
| Means for groups in homogeneous subsets are displayed. | | | |
| a. Uses Harmonic Mean Sample Size = 4.000. | | | |

| **Propionic** | | | |
| --- | --- | --- | --- |
| Tukey HSD^a^ | | | |
| Conditions | N | Subset for alpha = 0.05 | |
|  |  | 1 | 2 |
| DF_pH5 | 4 | .6500 |  |
| DF_pH7 | 4 |  | 2.9125 |
| EF | 4 |  | 4.2675 |
| Sig. |  | 1.000 | .254 |
| Means for groups in homogeneous subsets are displayed. | | | |
| a. Uses Harmonic Mean Sample Size = 4.000. | | | |

**PRODUCT YIELD**

| **Descriptives** | | | | | | | | | |
| --- | --- | --- | --- | --- | --- | --- | --- | --- | --- |
|  | | N | Mean | Std. Deviation | Std. Error | 95% Confidence Interval for Mean | | Minimum | Maximum |
|  |  |  |  |  |  | Lower Bound | Upper Bound |  |  |
| Acetic | EF | 4 | .2800 | .07118 | .03559 | .1667 | .3933 | .18 | .34 |
|  | DF_pH5 | 4 | .2725 | .12093 | .06047 | .0801 | .4649 | .10 | .37 |
|  | DF_pH7 | 4 | .3925 | .03202 | .01601 | .3416 | .4434 | .36 | .42 |
|  | Total | 12 | .3150 | .09453 | .02729 | .2549 | .3751 | .10 | .42 |
| Propionic | EF | 4 | .3025 | .10046 | .05023 | .1427 | .4623 | .17 | .39 |
|  | DF_pH5 | 4 | .1150 | .11030 | .05515 | -.0605 | .2905 | .02 | .25 |
|  | DF_pH7 | 4 | .4100 | .04243 | .02121 | .3425 | .4775 | .36 | .45 |
|  | Total | 12 | .2758 | .15090 | .04356 | .1800 | .3717 | .02 | .45 |

| **ANOVA** | | | | | | |
| --- | --- | --- | --- | --- | --- | --- |
|  | | Sum of Squares | df | Mean Square | F | Sig. |
| Acetic | Between Groups | .036 | 2 | .018 | 2.617 | .127 |
|  | Within Groups | .062 | 9 | .007 |  |  |
|  | Total | .098 | 11 |  |  |  |
| Propionic | Between Groups | .178 | 2 | .089 | 11.118 | .004 |
|  | Within Groups | .072 | 9 | .008 |  |  |
|  | Total | .250 | 11 |  |  |  |

| **Multiple Comparisons** | | | | | | | |
| --- | --- | --- | --- | --- | --- | --- | --- |
| Tukey HSD | | | | | | | |
| Dependent Variable | (I) Conditions | (J) Conditions | Mean Difference (I-J) | Std. Error | Sig. | 95% Confidence Interval | |
|  |  |  |  |  |  | Lower Bound | Upper Bound |
| Acetic | EF | DF_pH5 | .00750 | .05876 | .991 | -.1566 | .1716 |
|  |  | DF_pH7 | -.11250 | .05876 | .190 | -.2766 | .0516 |
|  | DF_pH5 | EF | -.00750 | .05876 | .991 | -.1716 | .1566 |
|  |  | DF_pH7 | -.12000 | .05876 | .158 | -.2841 | .0441 |
|  | DF_pH7 | EF | .11250 | .05876 | .190 | -.0516 | .2766 |
|  |  | DF_pH5 | .12000 | .05876 | .158 | -.0441 | .2841 |
| Propionic | EF | DF_pH5 | .18750^*^ | .06332 | .038 | .0107 | .3643 |
|  |  | DF_pH7 | -.10750 | .06332 | .258 | -.2843 | .0693 |
|  | DF_pH5 | EF | -.18750^*^ | .06332 | .038 | -.3643 | -.0107 |
|  |  | DF_pH7 | -.29500^*^ | .06332 | .003 | -.4718 | -.1182 |
|  | DF_pH7 | EF | .10750 | .06332 | .258 | -.0693 | .2843 |
|  |  | DF_pH5 | .29500^*^ | .06332 | .003 | .1182 | .4718 |
| *. The mean difference is significant at the 0.05 level. | | | | | | | |

| **Acetic** | | |
| --- | --- | --- |
| Tukey HSD^a^ | | |
| Conditions | N | Subset for alpha = 0.05 |
|  |  | 1 |
| DF_pH5 | 4 | .2725 |
| EF | 4 | .2800 |
| DF_pH7 | 4 | .3925 |
| Sig. |  | .158 |
| Means for groups in homogeneous subsets are displayed. | | |
| a. Uses Harmonic Mean Sample Size = 4.000. | | |

| **Propionic** | | | |
| --- | --- | --- | --- |
| Tukey HSD^a^ | | | |
| Conditions | N | Subset for alpha = 0.05 | |
|  |  | 1 | 2 |
| DF_pH5 | 4 | .1150 |  |
| EF | 4 |  | .3025 |
| DF_pH7 | 4 |  | .4100 |
| Sig. |  | 1.000 | .258 |
| Means for groups in homogeneous subsets are displayed. | | | |
| a. Uses Harmonic Mean Sample Size = 4.000. | | | |
